# Cardiotoxicity and cardiovascular disease risk assessment for patients receiving breast cancer treatment

**DOI:** 10.1186/s40959-017-0025-7

**Published:** 2017-10-17

**Authors:** Robyn A. Clark, Tania S. Marin, Narelle M. Berry, John J. Atherton, Jonathon W. Foote, Bogda Koczwara

**Affiliations:** 1 College of Nursing and Health Sciences, Flinders University, Adelaide, SA Australia; 2 Norwich Medical School, University of East Anglia, James Watson Road, Norwich Research Park, Norwich, NR4 7UQ UK; 3 Head Cardiology Royal Brisbane and Women’s Hospital, University of Queensland School of Medicine, Butterfield St & Bowen Bridge Rd, Herston, St Lucia, QLD Australia; 4 Department of Medical Oncology, Flinders Centre for Innovation in Cancer, Flinders University, Flinders Drive, Bedford Park, SA 5042 Australia

**Keywords:** (10) Cardiotoxicity, Risk assessment, Breast cancer, CVD risk factors, Heart health, Health literacy

## Abstract

**Background:**

Cardiotoxicity from anticancer therapy affects heart function and structure. Cardiotoxicity can also lead to accelerated development of chronic diseases, especially in the presence of risk factors.

**Methods:**

This study aimed to develop and pilot a combined cardiovascular disease and cardiotoxicity risk assessment questionnaire to quantify the potential extent of risk factors in breast cancer patients prior to treatment. The questionnaire underwent content and face validity evaluation by an expert panel followed by pilot testing in a sample of breast cancer patients (*n* = 36). Questionnaires were self-administered while attending chemotherapy clinic, in the presence of a research assistant.

**Results:**

Mean age of participants was 54.8 years (range 36–72 years). Participants reported CVD risk factors including diabetes 2.8%, hypertension 19.8%, hypercholesterolaemia 11% and sleep apnoea 5%. Lifestyle risk factors, included not eating the recommended serves of vegetables (100%) or fruit (78%) per day; smoking (13%) and regularly consuming alcohol (75%). Twenty five percent reported being physically inactive, 61%, overweight or obese, 24%, little or no social support and 30% recorded high to very high psychological distress. Participants were highly (75%) reluctant to undertake lifestyle changes; i.e. changing alcohol consumption; dietary habits; good emotional/mental health strategies; improving physical activity; quitting smoking; learning about heart-health and weight loss.

**Conclusion:**

This study is an important step towards prevention and management of treatment-associated cardiotoxicity after breast cancer diagnosis. We recommend that our questionnaire is providing important data that should be included in cancer registries so that researchers can establish the relationship between CVD risk profile and cardiotoxicity outcomes and that this study revealed important teaching opportunities that could be used to examine the impact on health literacy and help patients better understand the consequences of cancer treatment.

## Background

Advances in screening, early detection and treatment of cancer have led to improved survival of patients with cancer, but have also increased morbidity and mortality due to treatment side effects [1]. Cardiotoxicity from anticancer therapy is a direct effect of cancer treatment on heart function and structure and one of the most common toxicities of cancer treatment, leading to accelerated development of cardiovascular disease (CVD), especially in the presence of traditional cardiovascular risk factors [2]. Cardiotoxicity is particularly common in cancers treated with anthracyclines and radiation therapy, of which breast cancer is the most common [2].

Approximately 14,200 cases of breast cancer are diagnosed annually in Australia, with five and ten-year survival rates of 89% and 83% respectively [3]. A leading cause of mortality, breast cancer is primarily a disease of older women [4] and as the population ages, more women are likely to be diagnosed, and live longer, after treatment. Although breast cancer therapy is increasingly effective in improving tumour-free survival, this may not always translate into better overall survival because of treatment-related side effects, including cardiotoxicity. Indeed, in older women diagnosed with breast cancer (who are the majority of those diagnosed), mortality from heart failure now exceeds cancer-specific mortality after ten years [5]. Agents most frequently associated with cardiotoxicity include anthracyclines (1–26% of patients), high-dose cyclophosphamides (7–28%), trastuzumab (2–28%) and tyrosine kinase inhibitors (0.05–11%), all of which are frequently used in the treatment of breast cancer [5, 6]. Chest irradiation and many drugs used in breast cancer (e.g. anthracyclines) are implicated in this toxicity [6, 7]. Compounding this problem, 90% of Australians have at least one cardiac risk factor increasing their predisposition to toxicity. They have a seven-fold higher risk of heart failure death and a 15-fold higher risk of heart failure than the general population [5, 8]. The challenge is to tailor treatment to the molecular nature of the disease while simultaneously modifying its cardiac effects [6].

Cardiac clinicians routinely assess socio-demographic factors (e.g. level of education and access to health services) and manage psychosocial factors (e.g. depression) and lifestyle factors (e.g. diet, exercise and the patient’s ability to modify lifestyle) to reduce patients’ risk of cardiac disease. The authors, supported by recent literature, propose that these could be as important to consider as chemotherapy agent and dose in the assessment and management of cancer [5, 9].

To realise the potential of breast cancer treatments, cancer clinicians need assessment strategies that enable them to accurately identify treatment-related and pre-existing cardiac risks and to modify their combined potential to induce heart disease in breast cancer patients [10]. Given that a normal left ventricular ejection fraction at baseline cannot exclude the possibility of subsequent cardiotoxicity, serial cardiac imaging to assess left ventricular function is usually performed to identify patients who develop cardiotoxicity; however, outcomes are generally poor once a patient develops clinical heart failure [10].

The most recent European Society of Cardiology (ESC) position paper on cancer treatments and cardiovascular toxicity suggests that identifying high-risk patients prior to the administration of cancer therapy may allow treatment modifications to decrease the risk of subsequently developing cardiotoxicity [2]. However, this requires a better understanding of the predisposing factors for the development of CVD related to cancer treatment. We propose that a breast cancer-specific cardiotoxicity risk assessment, which stratifies patients to an individually tailored risk management plan, is warranted. Previous cardiotoxicity assessment tools have focused only on the factors with established causality (e.g. age, previous CVD, and chemotherapy or radiotherapy regime) [11–13]; however, currently there is no questionnaire that enables such a comprehensive CVD risk profile assessment specific to breast, or any other, cancer care [14].

## Methods

### Aim

The aim of this study was to produce a standardised, comprehensive approach to assessing CVD and cardiotoxicity risk before breast cancer treatment.

There were two objectives:

1. To develop and pilot test the risk assessment questionnaire; and
2. To obtain preliminary data on the extent and nature of modifiable and non-modifiable cardiovascular risk factors for cardiotoxicity in breast cancer patients.

### Design

This study was undertaken in two parts. Initially content development and face validity testing of a questionnaire by an expert panel to assess cardiotoxicity risk, based on the ESC position paper for cardiotoxicity baseline risk factors (Table 1). Following this, the questionnaire was piloted in a sample of breast cancer patients from two oncology clinics in Australia.

**Table 1:** Baseline risk factors for Cardiotoxicity (ESC Guidelines)

Current myocardial disease	Demographic and other CVD risk factors
• Heart failure (with either preserved or reduced ejection fraction)	Age (paediatric population < 18 years; >50 years for trastuzumab; >65 years for anthracyclines)
• Asymptomatic LV dysfunction (LVEF <50% or high natriuretic peptide^a^)	Family history of premature CV disease (<50 years)
• Evidence of CAD (previous myocardial infarction, angina, PCI or CABG, myocardial ischaemia)	Arterial hypertension
	Diabetes mellitus
	Hypercholesterolaemia
• Moderate and severe VHD with LVH or LV impairment	
• Hypertensive heart disease with LV hypertrophy	
• Hypertrophic cardiomyopathy	
• Dilated cardiomyopathy	
• Restrictive cardiomyopathy	
• Cardiac sarcoidosis with myocardial involvement	
• Significant cardiac arrhythmias (e.g.AF, ventricular tachyarrhythmias)	
Previous cardiotoxic cancer treatment	Lifestyle risk factors
• Prior anthracycline use	• Smoking
• Prior radiotherapy to chest or mediastinum	• High alcohol intake
	• Obesity
	• Sedentary habit

Summary of baseline risk factors for cardiotoxicity

*AF* atrial fibrillation, *CABG* coronary artery bypass graft, *CAD* coronary artery disease, *CV* cardiovascular, *LV* eft ventricule, *LVEF* left ventricular ejection fraction, *LVH* left ventricular hypertrophy, *VHD* valvular heart disease. B-type natriuretic peptide .100 pg/ml or N-terminal pro-B-type natriuretic peptide .400 pg/ml with no alternative cause

### Content development and face validity

A small working group of the expert panel members collated a battery of demographic, clinical and validated cardiovascular risk factor assessment tools (Table 2) to form the first draft of the questionnaire. The expert panel (*n* = 14) comprised representatives from the fields of medical oncology, cardiology, radiation oncology, dietetics, cancer nursing, psycho-oncology, pharmacy, exercise physiology, and two consumer representatives. All members volunteered to provide feedback on the draft questionnaire electronically in two rounds.

**Table 2:** Summary of published instruments used to evaluate cardiovascular risk factors

Data item	Measurement Reference	Question number
Household income categories	Australian Bureau of Statistics Cat 6523.0 Household Income and Wealth, Australia, 2013–141 [35]	3
Nutrition	Fruit and Vegetable Serves [32]	5
Alcohol consumption	Australian guidelines to reduce health risks from drinking alcohol [27]	17
Physical activity	The Godin-Shephard leisure-time physical activity questionnaire [21]	18–21
Emotional health	Kessler Psychological Distress Scale (K10) [18]	23
	MMOS_SS [19]	
Cardiac Health	York Cardiac Beliefs Questionnaire [25]	24
Sleep	Simplified model of screening questionnaire and home monitoring for obstructive sleep apnoea in primary care [33]	37
Body Mass Index and Waist circumference	National Health and Medical Research Council (2013) Clinical practice guidelines for the management of overweight and obesity in adults, adolescents and children in Australia [34]	12

Summary of questionnaires used to evaluate risk factors

In Round 1, panel members were asked to provide unstructured feedback on all aspects of the preliminary questionnaire. Their qualitative responses were synthesised, reduced and categorised to further modify the questionnaire. The modified questionnaire underwent two further electronic consensus rounds. These rounds focussed on ensuring that all the important items were incorporated; that there was no overlap or ambiguity of items; that items were scored appropriately; and that the questionnaire was relevant to end-users. In addition, the expert panel members were asked to complete a content validity index (CVI) on each question or section (e.g. pre-existing tool). The CVI was a four-point Likert scale that assessed the clarity of each item used, wherein 1 = very unclear, 2 = unclear, 3 = clear, 4 = very clear.

### Pilot testing questionnaire

#### Setting

The questionnaire was pilot tested for feasibility and acceptability by thirty-six patients from two Australian public facility oncology clinics, one in South Australia and the other in Queensland, between October 2015 and March 2016. As this was a pilot study using a battery of validated instruments, a power calculation was not applicable. The sample size of thirty-six was based on feasibility, time and funding. It is intended that outcomes from this study will be used to generate sample size calculations for future large cohort testing of the questionnaire.

#### Consumer involvement

This team of investigators recognises that consumers’ experience of breast cancer, their knowledge of the motivations and capacities of consumers to engage with research, coupled with their awareness of consumers’ needs, were integral to the success of this initiative. We consulted the Breast Cancer Network of Australia (BCNA) and the Australian National Heart Foundation in developing this project. We did so because the consumer representatives of these organisations are supported and trained to engage meaningfully in the research context and to advocate for consumers with different experiences to their own. Consumers and policy advisors from both organisations kindly contributed valuable advice about the content, relevance and feasibility of this study, and the BCNA consumer representatives agreed to be named as co-investigators.

#### Participants

Inclusion criteria for participants were: a new diagnosis of breast cancer; referral for chemotherapy and/or radiation therapy, up to third chemotherapy cycle; and able to provide informed consent. Exclusion criteria were: beyond third chemotherapy cycle; and unable or unwilling to give consent, or participate.

### Recruitment and consent

Eligible patients were selected by the clinical oncologist at each site and introduced to the research assistants (RAs). Once patients had been identified, they were approached during their waiting period on, or before, the third session of chemotherapy/radiotherapy treatment and invited to participate in a research survey about cardiotoxicity. Patients were given the participant information sheet and consent form to read, either at that time, or later in their own time. Once consent was received, the participant was given the questionnaire to complete while they waited for chemotherapy. An RA from the team was present to answer any questions that may have arisen, and to collect the data once the respondent had completed the questionnaire.

### Data sources

Data were sourced from the medical records of participants for demographics and clinical information (such as diagnosis and chemotherapy regime), as well as self-reported data from the tools within the questionnaire.

### Variables

#### Cardiac risk factors

As per National Heart Foundation guidelines [15], items collected included smoking, high blood cholesterol and high blood pressure; history of cardiovascular disease, diabetes, age ≥ 65 years, ethnicity, family history of cardiac disease and body mass index (BMI) [16]. Other non-modifiable risk factors included education level, postcode, income level, and private health insurance. These questions were taken directly from the Australian Bureau of Statistics (ABS) census [17]. All of these risk factors can be compared to the prevalence within the total Australian population.

#### Cancer and cancer treatment-related factors

Items included were: cumulative anthracycline dose ≥550 mg—particularly bolus administration—alone or in conjunction with infusional 5-fluorouracil, capecitabine, or taxanes; high-dose cyclophosphamide; aromatase inhibitor or tamoxifen therapy; mTOR inhibition; HER-2 blocking agents, e.g. trastuzumab; anti-angiogenic agents, e.g. bevacizumab; chest irradiation; and co-morbidity of blood cancer [2].

#### Psychosocial factors

Psychosocial factors were measured using the Kessler Psychological Distress Scale (K10) [18] and the eight-item modified Medical Outcomes Study Social Support Survey (MMOS-SS) [19]. Cardiac health behaviour, ability, willingness, knowledge and attitudes (ordinal and Likert-scale data) and standard items from the ABS Australian Health Survey (AHS) [20] elicited patients’ modifiable health practices, such as food choices, smoking, and alcohol consumption. Physical activity was measured with the Godin Leisure TimE Questionnaire (GLTEQ) [21] and exercise practices were asked as per the current unhealthy CVD risk factor and lifestyle profile of the general Australian population [22, 23]. These AHS items enabled comparison with national rates for these activities. Fruit and vegetable consumption was measured using two items from the South Australian Monitoring and Surveillance System (SAMSS) [24]. To gain insight into the participant’s health literacy, knowledge and beliefs around heart disease and its treatment, we included the York Cardiac Beliefs Questionnaire [25].

### Patient content validation

Participants were asked four additional questions to ascertain their opinion of the questionnaire items: 1) whether the items were easy to understand; 2) whether the items were relevant; 3) whether the format was acceptable; and 4) whether the time taken to complete the assessment was acceptable.

### Statistical analysis

Data were manually entered from the questionnaires into Microsoft Excel and a 10% quality data assessment was completed for accuracy. Missing data were examined for extent, pattern, and randomly or systematically missing. Depending on the potential source of bias entailed in the missing data, an appropriate method (e.g. item deletion or data imputation) was then used. All data were then imported into Statistical Package for the Social Sciences (SPSS) for Windows, Version 20.0 [26], for analysis.

Participant characteristics and clinical data are presented as frequency and summary descriptive statistics. Continuous variables have been reported as means and standard deviations for normally distributed data. Proportions summarise categorical variables. Comparability of treatments received as per recruitment pathways have been evaluated for participants’ demographic and medical characteristics using cross-tabulations.

## Results

### Questionnaire content development and validity testing

The expert panel assessed the questionnaire in terms of item relevance using the Likert scales for each section with a mean CVI per item of at least three needed to be accepted. If not, items were modified through further expert panel consultation or deleted, after which consensus was obtained on the final version of the questionnaire. The final questionnaire contained 19 pages and 355 items or variables (151 from clinical notes and 204 self-reported). These covered demographics, clinical history, cancer treatment, and validated tools to measure current cardiovascular risk factors, cardiac disease and health literacy.

### Pilot test

The pilot study aimed to recruit twenty participants from each site for a total of forty participants. Final recruitment was thirty-six on completion of the recruitment period.

### Characteristics of participants

A summary of the characteristics, cancer diagnoses and treatment, and cardiovascular risk factors are presented in Table 3 and where possible have been compared to Australian norms. The mean age of participants was 54.8 years compared to the national average for women, which was 39.4 years. Within the group, 5.6% identified as being of Aboriginal and/or Torres Strait Islander descent compared to the national reported 2.5%. One patient had a family history of myocardial infarction. Participants reported CVD risk factors including diabetes 2.8%, hypertension 19.8%, hypercholesterolemia 11%, and sleep apnoea 5%.

**Table 3:** Summary of Cancer and Cardiovascular Risk Factors

Risk Factors	Participants *n* = 36	Australian Population
	*n* (%)	%
Mean age years (Female) Range (36–72 years)	54.8	39.4
Sex (Female)	36 (100)	50.3
Indigenous status	2 (5.6)	3.1
Usual residence -Metropolitan area	27 (75)	68.5
Family History Type 2 Diabetes	0 (0)	–
Family History Hypertension	0 (0)	–
Family History Hypercholesterolaemia	1 (2.8)	–
Family History Myocardial Infarction	1 (2.8)	–
Patient History Type 2 Diabetes	1 (2.8)	4.6
Patient History Hypertension	7 (19.5)	21.5
Patient History Hypercholesterolaemia	4 (11.1)	32.3
Patient History Previous History Myocardial Infarction	0 (0)	3.4
Eats recommended 5 vegetable serves per day	0 (0)	8
Eats recommended 2 fruit serves per day	22 (62.9)	48
Current smoker	5 (13.9)	16
Alcohol intake (at least once a week)	8 (22.2)	37.7
Moderately active or active physically	27 (75)	44
Little or no social support	8.7 (24.2)	–
High to very high K10 score (22–50)	8 (29.6)	10.8
Earnings below the total gross mean Australian annual household income	26 (72.2)	80
Earnings above the total gross mean Australian annual household income	3 (8.3)	20
Household income: Prefer not to answer	7 (19.4)	0
Sleep		
Mean hours of sleep per night	6.7 (NA)	7.3
Snoring (regular)	13 (36.1)	–
Sleep apnoea diagnosis	2 (5.7)	4.0
Body Mass Index (BMI >25 kg/m2 Overweight)	22 (61.1)	63
Waist circumference ≥ 80 cm (Heart Foundation recommendation for women)	4 (100)	79.4

Demographic and clinical characteristics of participants

The lifestyle CVD risk factor questions revealed that no participants were eating the recommended five serves of vegetables per day and 22% ate the recommended two serves of fruits per day [27]. Thirteen percent reported being current smokers and 75% regular consumers of alcohol. Additionally, 25% reported being physically inactive, 24% had little to no social support and 30% recorded high to very high psychological distress. The participants reported sleeping less than the national average (6.7 h compared to 7.3 h per night), and 61 % of participants were overweight (BMI ≥ 25 kg/m^2^).

### Cardiotoxicity risk factors

Details of known risk factors for cardiotoxicity from cancer treatment are detailed in Table 1 [2]. In this small group of breast cancer patients there was a high potential for cardiotoxicity with 76% of the participants being over fifty years of age, 30% having pre-existing CVD or other chronic diseases, and only one third of participants having had a prior electrocardiogram or a prior echocardiogram (Table 4) [2].

**Table 4:** Summary of participant cardiotoxicity risk factors

Characteristics (*n* = 36)	Participants *n* (%)	
Age > 50 years	27 (76)	
Cancer type		
Breast	22 (61.2)	
Breast left	2 (5.6)	
Carcinoma of breast (elective (L) mastectomy 4/8/15)	1 (2.8)	
Lower-inner quadrant of breast	1 (2.8)	
Malignant breast cancer	1 (2.8)	
Malignant neoplasm of breast	3 (8.3)	
Malignant neoplasm of upper-outer quadrant of breast	1 (2.8)	
Metastatic HER-2 pos breast cancer	1 (2.8)	
Palpable cancer right breast	1 (2.8)	
Missing	3 (8.3)	
Chemotherapy		
Mean number of chemotherapy cycles 4.9 (Median 3) Range (1–16)		
Agent class	Mean Dose (mg)	n (%)
Alkylating agents		
Carboplatin	658	5 (13.8%)
Cyclophosphamide	1073	20 (56%)
Anthracyclines		
Doxorubicin	110	11 (30%)
Antimetabolites		
Fluorouracil	996	6 (17%)
Mitotic Inhibitors		
Docetaxel	158	15 (42%)
Paclitaxel	156	20 (56%)
Monoclonal Antibody		
Denosumab	120	1 (2%)
Trastuzumab	493	13 (36%)
Baseline Cardiac Assessment		
Echocardiography	14 (38.9)	
Left Ventricular Ejection Fraction <45%	0 (0)	
Angiography	1 (2.8)	
Electrocardiogram	11 (30.6)	

Summary of cardiotoxicity risk factors

### Knowledge of heart disease

Knowledge and beliefs of heart disease are presented in Table 5. There was considerable misconception (25.5%) or no knowledge (6.9%) about heart disease within this group.

**Table 5:** York Angina Knowledge and Beliefs

Cardiac Health (Respondents *n* = 36)	Incorrect belief *n* (%)	No Knowledge *n* (%)
One of the main causes of heart disease is stress	23 (63.9)	2 (5.6)
Heart problems will definitely shorten your life whatever age you are	21 (58.3)	2 (5.6)
Angina is a kind of small heart attack	19 (54.3)	4 (11.1)
Once you have had one heart attack you are bound to have another one	18 (50)	3 (8.3)
A heart attack makes a weak area in the heart wall that can easily rupture	16 (44)	8 (22.2)
People who have heart problem should always avoid stress	13 (36.1)	3 (8.3)
It’s okay to disagree with people with heart problems	12 (33.3)	2 (5.6)
It is dangerous for people who have heart problems to argue	12 (33.3)	2 (5.6)
People with heart disease should take life easy	11 (30.6)	3 (8.3)
People develop heart disease because of worry in their life	10 (27.8)	2 (5.6)
Any sort of excitement could be bad if you have heart problems	9 (25.0)	3 (8.3)
It’s a good idea to check to see how you feel before doing something	9 (25.0)	3 (8.3)
Heart problems are a sign that you have a worn out heart	9 (25.0)	4 (11.1)
It is important to avoid anything that might bring on angina or chest pain	9 (25.0)	2 (5.6)
Rest is the best medicine for heart problems	9 (25.0)	2 (5.6)
People who have heart problems should never get excited or upset	8 (22.2)	2 (5.6)
Your heart is like a battery, the more you do, the faster it runs down	7 (19.4)	3 (8.3)
People with heart problems should live life to the full	5 (13.9)	3 (8.3)
There’s not much you can do about heart problems	4 (11.1)	2 (5.6)
Heart problems are often caused by peoples’ lifestyle	4 (11.1)	6 (16.7)
Doing exercise can strengthen the heart muscle	2 (5.6)	3 (8.3)
It is important for people with heart problems to carry on doing enjoyable things	2 (5.6)	2 (5.6)
You can reduce your risk of more heart problems	1 (2.8)	1 (2.8)
Changing your lifestyle can reduce your risk of more heart problems	0 (0)	2 (5.6)
Mean Incorrect Beliefs and No knowledge (%)		
	25.5%	6.9%

Heart health knowledge and beliefs

### Willingness to modify risk factors and make lifestyle changes

One of the final questions in the questionnaire asked if participants would be interested in receiving further information or support about risk factor and/or lifestyle modification (see Table 6). Generally there was a high level of unwillingness to undertake lifestyle changes with 88% stating they were not prepared to modify alcohol consumption; 44% unwilling to modify dietary habits; 72% not interested in receiving good emotional/mental health strategies; 61% not willing to improve physical activity; 68% of smokers unwilling to receive information about quitting; and 50–78% not interested in learning more about heart health or losing weight.

**Table 6:** Willingness to modify risk factors or make lifestyle changes

Cardiovascular and Cancer Risk Factors	^a^Unwilling to make lifestyle changes n (%)
Alcohol consumption	16 (88.9)
Dietary habits	16 (44.4)
Good emotional/mental health strategies	26 (72.2)
Exercising/physical activity	22 (61.1)
Quit smoking (smokers)	4 (66.7)
Understanding heart health	28 (77.8)
Weight loss or gain	18 (50)

Willingness to make lifestyle changes

^a^Data only from participants who had the nominated risk factors

### Completion of questionnaire

The average time taken for participants to complete the questionnaire was 25 min. Times ranged from 20 to 45 min. Participants reported both positive and negative views. Two recurring themes were confusion about why we were talking about heart issues in the cancer clinic, and the time required to complete the questionnaire.

### Other feedback and comments

In the section where participants were asked to give feedback on the questionnaire, 75% of participants took the opportunity to give feedback. Positive comments included saying that the questions were well worded or suggesting relevant question improvements. Negative comments related to why we were asking about income and why we were asking questions about the heart in a chemotherapy clinic.

## Discussion

Previous cardiotoxicity assessment tools have focused only on factors with established causality e.g. age, previous CVD, and chemotherapy or radiotherapy regime [11–13]. The aim of this study was to produce a standardised, comprehensive approach to assessing the risk of cardiotoxicity, which included assessment and measurement of CVD risk and lifestyle factors that may indicate undiagnosed CVD prior to cancer treatment. From a nursing point of view, as many risk factors for cancer and CVD are the same, this questionnaire could create a ‘teaching moment’ and an opportunity to introduce risk management and lifestyle education as part of cancer treatment [28, 29]. Future studies exploring the role of patient education as a strategy to change behaviour and increase engagement in care are warranted.

During questionnaire development, there were two important findings. Firstly, the questionnaire was large (19 pages with 355 items) and took an average of twenty-five minutes to complete. It is recommended that further work be carried out to shorten the questionnaire before this data collection tool is used again. There was a large variation in the waiting times within the two pre-chemotherapy clinics and some participants became distressed at needing to complete such a large document while waiting to be called to chemotherapy. Others needed to take the questionnaire home to complete. In this case, they were supplied with a reply paid envelope to return to us by mail. An alternative approach may be to administer questionnaires for cardiotoxicity risk assessment while waiting for routine echocardiography prior to cancer treatment; however, only one third of the patients in our study reported having an echocardiogram prior to chemotherapy. Whilst this may have been under-reported, this would still be unlikely to result in a comprehensive capture. Given that cardiac imaging is performed by different service providers, this further complicates the data collection.

Secondly, many participants asked the research team why they were being asked about their heart health in the breast cancer chemotherapy clinic. This was a positive finding for the research as it indicated to our team that we had created a teaching opportunity.

In the pilot testing, as expected, we found an older group of women than the mean national age (54.8 years compared to 39.4 years national average). According to the ESC guidelines, age is a risk factor for cardiotoxicity particularly for those fifty years and over. The diagnoses of the participants indicated that they would be receiving potentially cardiotoxic chemotherapy and each patient’s regimen comprised individualised composites and doses of the cytotoxic agents. There was evidence of pre-existing risk factors for CVD in 30% of participants and 61% were overweight or obese reflecting the current CVD risk factor profile of all Australians [22, 23]. Participants reported a strong unwillingness to undertake lifestyle changes. We acknowledge that some of the unwillingness would have been related to a wish to deal with the cancer issues and possibly an inability to do things like physical activity due to the acute effects of treatment (e.g. nausea and lethargy). However, our results would indicate an opportunity for cardio-oncology educational interventions similar to models that are being researched internationally [28, 30, 31].

### Relevance to breast cancer patients

The outcomes of this study are relevant to all cancer patients and cancer survivors who have received potential cardiotoxic cancer treatment and the authors stress the importance of a cardio-oncology co-management of patients with an increased burden of risk factors. Australian health services do not currently undertake standardised, comprehensive cardiac risk assessments in breast cancer patients, nor do they provide structured health promotion programs to support breast cancer consumers to minimise lifestyle-related cardiac risk during and after treatment, despite the public and personal health benefits that these would provide. A range of survivorship programs have been implemented in recent years in recognition of the chronic nature of cancer; however, most targeted specific symptoms and were not grounded in sound chronic disease self-management principles and the benefits, not only for heart health but effects on cancer recurrence [2]. They are also not tailored to the distinct needs of breast cancer survivors, such as difficulties engaging in exercise after breast reconstruction and body image issues [9]. As the field of cardio-oncology grows and develops, it is hoped that the collaboration between cancer and cardiology clinicians will facilitate a comprehensive and seamless approach to care for cancer patients at risk of heart disease.

### Implications for translation to practice and further research

This pilot study provides preliminary data for an effectiveness trial to measure the impact of CVD profiling and cardiotoxicity risk assessment, to provide an appropriate clinical pathway to manage patients safely through their cancer treatment. The project team will utilise a ‘bottom up’ approach to drive the translation of this approach into practice, working directly with our established network of consumers, clinicians and key cancer organisations to ensure their engagement. We will demonstrate the personal, clinical and cost-effectiveness benefits of our approach, with the goal being to increase both consumer and clinician awareness and understanding of cardiotoxicity.

### Limitations

This study was a pilot and generalisability is limited. The results of the study have provided important lessons about the size, timing, design, location and mode of delivery of the data collection. This was not a random sample of participants and there may have been bias in sampling as oncologists may have chosen patients who were at a higher literacy level, or who were coping better with chemotherapy, than other potential participants. This may have led to selection bias.

### Future research

Valuable lessons will be implemented in the next phase of our research. The opportunity to expand on the teaching opportunities revealed during this pilot study could be used to examine impact on health literacy and help patients better understand the consequences of cancer treatment.

## Conclusion

This study is an important first step towards evidence-based and personalised assessment, prevention and management of treatment-associated cardiotoxicity after breast cancer. Important teaching opportunities revealed during this pilot study could be used to examine the impact on health literacy and help patients better understand the consequences of cancer treatment. We recommend that our questionnaire is providing important data that should be included in cancer registries so that researchers can establish the relationship between CVD risk profile and cardiotoxicity outcomes.

## Abbreviations

ABS: Australian Bureau of Statistics
AHS: Australian Health Survey
BCNA: Breast Cancer Network of Australia
BMI: Body Mass Index
CVD: Cardiovascular disease
CVI: Content Validity Index
ESC: European Society of Cardiology
GLTEQ: Godin Leisure TimE Questionnaire
HREC: Human Research Ethics Committee
K10: Kessler Psychological Distress Scale
MMOS-SS: Medical Outcomes Study Social Support Survey
RA: Research Assistant
SACHREC: Southern Adelaide Clinical Human Research Ethics Committee
SAMSS: South Australian Monitoring and Surveillance System
SPSS: Statistical Package for the Social Sciences
